# The Influence of Mesenteric Defects Closure on the Use of Computed Tomography for Abdominal Pain 5 Years After Laparoscopic Gastric Bypass—a Post Hoc Analysis of a Randomized Clinical Trial

**DOI:** 10.1007/s11695-021-05778-z

**Published:** 2021-11-23

**Authors:** Demir Amanda, Påhlson Elin, Norrman Eva, Erik Stenberg

**Affiliations:** 1 Department of Surgery, Faculty of Medicine and Health, Örebro University, 701 82 Örebro, Sweden; 2 Department of Medical Physics, Faculty of Medicine and Health, Örebro University, Örebro, Sweden

**Keywords:** Randomized clinical trial, Humans, Gastric bypass, Laparoscopy, Obesity, Internal hernia, Small bowel obstruction, Radiation, Computed tomography, Mesenteric defects

## Abstract

**Background:**

Abdominal pain after laparoscopic Roux-en-Y gastric bypass (LRYGB) is a common and unwanted complication that typically leads to further exploration through radiology. Concerns have been raised regarding the consequences of this radiation exposure and its correlation with the lifetime risk of cancer. The aim of this study was to evaluate the differences in computed tomography (CT) use between LRYGB patients with open and closed mesenteric defects and to assess the radiological findings and radiation doses.

**Methods:**

This subgroup analysis included 300 patients randomized to either closure (*n* = 150) or nonclosure (*n* = 150) of mesenteric defects during LRYGB. The total number of CT scans performed due to abdominal pain in the first 5 postoperative years was recorded together with the radiological findings and radiation doses.

**Results:**

A total of 132 patients (44%) underwent 281 abdominal CT scans, including 133 scans for 67 patients with open mesenteric defects (45%) and 148 scans for 65 patients with closed mesenteric defects (43%). Radiological findings consistent with small bowel obstruction or internal hernia were found in 31 (23%) of the scans for patients with open defects and in 18 (12%) of the scans for patients with closed defects (*p* = 0.014). The other pathological and radiological findings were infrequent and not significantly different between groups. At the 5-year follow-up, the total radiation dose was 82,400 mGy cm in the nonclosure group and 85,800 mGy cm in the closure group.

**Conclusion:**

Closure of mesenteric defects did not influence the use of CT to assess abdominal pain.

**Graphical abstract:**

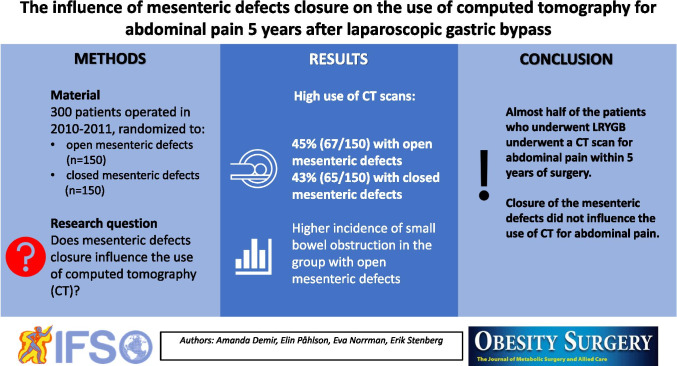

## Introduction

Obesity is associated with a wide range of negative health effects such as sleep apnea, hypertension, depression, diabetes, cardiovascular disease, and cancer [1]. This condition is a rapidly increasing concern of the modern world, and surgical treatment, with gastric bypass as a viable option, is currently the best way to achieve long-term results [2]. However, similar to all surgical interventions, this treatment is not without risks for complications.

Acute, intermittent, and chronic abdominal pain after laparoscopic Roux-en-Y gastric bypass (LRYGB) is a common complication that has been reported to require healthcare contacts for as many as 1 out of 3 patients after gastric bypass surgery [3, 4]. The evaluation often presents as a diagnostic challenge, in particular for those unfamiliar with the anatomic construct of RYGB.

The causes of abdominal pain in this population are diverse and range from benign to potentially life-threatening conditions. Small bowel obstruction (SBO) due to internal hernia (IH) is a particularly sinister condition that has become a major concern over the past decades. If no measures are taken to reduce the risk of SBO, the incidence may be as high as 10%, of which IH is the predominant cause [5, 6].

Acute incarceration of an IH can cause necrosis and a significant loss of small bowel with a nonnegligible risk for mortality [7]. While routine closure of mesenteric defects has been reported to successfully reduce the risk of IH, SBO and IH still occur. Furthermore, mesenteric defect closure is associated with an increased risk for obstruction of the jejunojejunal anastomosis, resulting in bowel obstruction or relative obstruction with chronic pain [5].

When confronted with abdominal pain in LRYGB patients, physical examination alone is often inconclusive, and the results are hard to interpret. Clinical assessments are therefore often combined with radiological examinations. Currently, computed tomography (CT) is the preferred modality, with a reported sensitivity of 83–85% [5, 8].

The use of various medical imaging examinations has expanded in the last couple of years, with an increase outpacing that of all other categories of physician services except laboratory tests [9, 10]. Considerable concerns have now been raised regarding the consequences of the associated radiation exposure and, most importantly, its contribution to the lifetime risk of cancer [11–13]. It has been estimated that approximately 0.2–2% of incident cancers could be attributable to CT scans [14].

The aim of the present study was to investigate if there is a difference in the use of CT for abdominal pain depending on whether the mesenteric defects are closed during LRYGB and to assess the radiological findings and radiation doses.

## Methods

### Study Design

This study represents a subgroup analysis of patients included from one surgical center in an original multicenter randomized clinical trial (RCT) comparing mesenteric closure with running nonabsorbable sutures to nonclosure during LRYGB surgery. The enrollment, inclusion, and randomization processes have previously been reported in detail [5]. In brief, oral and written consent was obtained from all patients. All patients were randomly assigned intraoperatively to mesenteric defect closure or nonclosure in a 1:1 ratio, with permuted blocks of different sizes, stratified by center. For the present study, surgery was performed exclusively at Örebro University Hospital and a Lindesberg County Hospital (one center at two locations) between 1 May 2010 and 14 November 2011.

All CT scans acquired for any symptoms of abdominal pain within the first 5 postoperative years were obtained from the regional hospital database and collected together with the indications for the examination, radiological findings, and radiation exposure, as measured by dose-length product (DLP, in mGy cm) and computed tomography dose index (CTDI, in mGy). Follow-up imaging performed due to the findings on the primary scan was included, as they were also considered a consequence of abdominal pain. Scans acquired to assess multiple traumas were excluded. The effective dose (E) was calculated using a conversion factor (k) specific for the anatomic region: E = k*DLP [15]. The primary outcome was the difference in CT use between patients with open and closed mesenteric defects. The secondary outcomes of interest were the nature of the radiological findings and the total amount of radiation exposure in each group.

### Statistical Analysis

The power calculation for the main study has been presented previously. For the present study, a power calculation was conducted assuming a risk reduction in CT usage equivalent to the described reduction in risk for small bowel obstruction. To detect a difference with a power of 80% at the 5% significance level, a minimum of 108 patients were needed in each group.

The results were analyzed on an intention-to-treat basis. Variables were compiled with standard descriptive statistics and presented in terms of frequency, mean, standard deviation, and/or percentage, if suitable. The study arms were compared using independent-samples *t* tests for continuous numeric variables with a normal distribution and Mann–Whitney *U* tests for data without a normal distribution. A skewness between − 0.5 and 0.5 was considered a normal distribution. Pearson’s *χ*2 test was used for dichotomous nominal variables. Statistical significance was set at *p* < 0.05, and two-tailed tests were applied. All data analysis was performed using IBM SPSS Statistics version 25.0 (SPSS, Chicago, IL, USA).

The trial is registered in ClinicalTrial.gov, number NCT01137201.

### Ethics

The study was approved by the Regional Ethics Board (DNR:2009/415/3) and followed the standards of the 1964 Helsinki declaration and its later amendments.

## Results

**Fig. 1:**
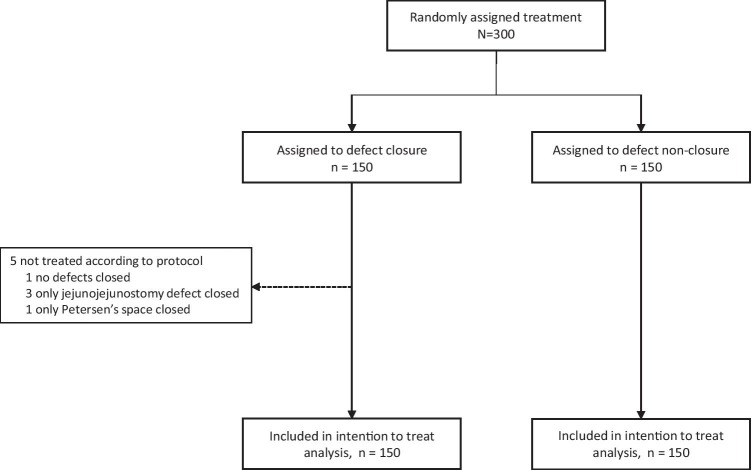
CONSORT diagram

During the inclusion period, 300 patients agreed to participate in the study and were randomized in equal proportions for either mesenteric defect closure using running nonabsorbable sutures or nonclosure. Five patients in the group randomized to nonclosure of the mesenteric defects did not fully receive the allocated intervention: one had neither of the mesenteric defects closed (due to adhesions), 3 had only the defect beneath the jejunojejunostomy closed, and 1 had only the defect at Petersen’s space closed. All of these patients were included in the analyses in accordance with the intention-to-treat principle (Fig. 1).

There were no significant differences in baseline characteristics between the groups (Table 1).

**Table 1:** Demographic data and patient characteristics

	Open defects	Closed defects
Male sex, *n* (%)	54 (36.0%)	44 (29.3%)
Age (years), mean ± SD	43 ± 10.5	44 ± 11.4
Preoperative BMI (kg/m^2^), mean ± SD	42.2 ± 5.0	42.3 ± 4.7
Active smokers, *n* (%)	30 (20.0%)	21 (14.0%)
Hypertension, *n* (%)	48 (32.0%)	44 (29.3%)
Diabetes, *n* (%)	25 (16.7%)	31 (20.7%)
Sleep apnea, *n* (%)	17 (11.3%)	16 (10.7%)
Depression, *n* (%)	16 (10.7%)	14 (9.3%)
Previous cholecystectomy, *n* (%)	20 (13.3%)	21 (14.0%)

*SD*, standard deviation; *Preop*, preoperative; *BMI*, body mass index

### Use of CT Scans

During the study period, the participants underwent a total of 285 abdominal CT scans. After the exclusion of 4 scans for multiple traumas, 281 CT scans were acquired for 132 patients based on the indication of abdominal pain (incident rate ratio = 0.19 examinations/person year). Some 44.7% of patients in the nonclosure group (*n* = 67) were examined with at least one CT scan, compared to 43.3% of patients with closed mesenteric defects (*n* = 65, *p* = 0.816). The highest number of scans per patient was 7 in the nonclosure group and 16 in the group randomized to mesenteric defect closure. There was no significant difference regarding the total number of postoperative CT scans between the two groups (Table 2).

**Table 2:** Number of CT scans due to abdominal pain within the first 5 years after surgery

	Open defects	Closed defects	*P*
Patients with no CT scans, *n* (%)	83 (55.3%)	85 (56.7%)	Ref
Patients with 1 CT scans, *n* (%)	38 (25,3%)	36 (24.0%)	0.780
Patients with ≥ 2 CT scans, *n* (%)	29 (19.3%)	29 (19.3%)	0.938
Total number of scans^1^	133	148	N/A

*CT*, computed tomography; *LRYGB*, laparoscopic Roux-en-Y gastric bypass; *N/A*, not applicable

^1^Each patient could be scanned more than once

CT signs of SBO and IH were more common in the group with open mesenteric defects (23.3% versus 12.2%). Twelve patients with open mesenteric defects suffered from SBO compared to 3 with closed defects (*p* = 0.017). The other findings explaining the pain were infrequent and not significantly different between groups. For the majority of patients, no radiologic explanations were found for their abdominal pain (Table 3). Twelve patients underwent a diagnostic laparoscopy (with additional gastroscopy in the case of negative findings) despite a negative CT. In 8 patients, no pathology was found. For the remaining operations, herniated bowel without other signs of obstruction was found beneath the jejunojejunostomy on 1 occasion, a distal ileitis on 1 occasion, and 2 patients had a marginal ulcer.

**Table 3:** Findings on CT scans acquired for abdominal pain within the first 5 years after surgery

	Open defects	Closed defects	*P*
Small bowel obstruction/internal hernia, *n* (%)	31 (23.3%)	18 (12.2%)	0.014
Normal examination/no explanation of the pain, *n* (%)	59 (44.4%)	75 (50.7%)	0.290
Other findings explaining the pain, *n* (%)	43 (32.3%)	55 (37.1%)	0.396
Ureteral stone	10 (7.5%)	2 (1.4%)	
Diverticulitis	4 (3.0%)	9 (6.1%)	
Incisional hernia	3 (2.3%)	5 (3.4%)	
Cholecystitis	3 (2.3%)	2 (1.4%)	
Gallstone	2 (1.5%)	5 (3.4%)	
Appendicitis	1 (0.8%)	3 (2.0%)	
Choledocholithiasis	1 (0.8%)	3 (2.0%)	
Postoperative bleeding	2 (1.5%)	2 (1.4%)	
Miscellaneous	17 (12.8%)^1^	24 (16.2%)^2^	

*CT*, computed tomography; *LRYGB*, laparoscopic Roux-en-Y gastric bypass

^1^Umbilical hernia *n* = 2, colitis *n* = 2, obstipation *n* = 2, ovarian cyst *n* = 2, endometritis *n* = 1, intestinal invagination *n* = 2, abscess *n* = 1, vertebral compression fracture *n* = 1, inguinal hernia *n* = 1, costal fracture *n* = 1, gastrointestinal perforation *n* = 1, pyelonephritis *n* = 1

^2^Umbilical hernia *n* = 2, colitis *n* = 1, obstipation *n* = 1, ovarian torsion *n* = 1, endometriosis *n* = 2, subcutaneous hematoma *n* = 5, tumor *n* = 5, diverticulosis *n* = 3, pulmonary pathology *n* = 2, bile leakage *n* = 1, anastomotic stenosis *n* = 1

### Radiation Exposure

The total radiation dose measured as DLP was 168,200 mGy cm for the entire cohort, with a distribution of 82,400 mGy cm in the nonclosure group and 85,800 mGy cm in the closure group. The mean CTDI during examinations was 10.3 ± 4.71 mGy with a mean radiation dose per patient for those with open mesenteric defects of 500 mGy cm ± 1100 (DLP) and for those with closed mesenteric defects 10.5 ± 5.41 mGy during examinations with a mean radiation dose of 600 mGy cm ± 1100 (DLP) per patient (*p* = 0.909). The average effective dose was estimated to be 9 mSv.

## Discussion

In this post hoc analysis of a subgroup of patients randomized to mesenteric defect closure or nonclosure, no significant difference in terms of CT use for abdominal pain or radiation exposure during the first 5 postoperative years after surgery was seen.

In all, 43% of patients with closed mesenteric defects and 45% of those without closure underwent at least one abdominal CT scan to evaluate abdominal pain. These numbers are well in line with those reported previously for patients who underwent laparoscopic gastric bypass surgery [16, 17]. While SBO and IH were more common among patients with open mesenteric defects, the total number of abdominal CT scans was not lower for patients with closed defects. This finding confirms the secondary analyses of previous studies, reporting no difference in bodily pain or postoperative pain, as estimated using visual analog scales [18, 19]. While closure of mesenteric defects reduces both acute and more intermittent presentations of IH, it has been associated with an increased risk for relative obstruction and kinking of the jejunojejunostomy [5].

The large number of cases of abdominal pain in which no explanation could be seen on abdominal CT conforms with the high prevalence of chronic abdominal pain as well as irritable bowel syndrome after gastric bypass surgery [4]. In addition, several studies have previously reported neuropathy as a common complication of bariatric surgery, as well as postoperative pain. While this condition is common and often related to poor nutritional status, it is chiefly peripheral, and no research has specifically examined abdominal manifestations following mesenteric defect closure [20].

The large number of patients evaluated with CT scans and the large number of scans for certain patients may reflect the current practice for evaluating patients with abdominal pain who have previously undergone gastric bypass surgery. These patients generally present abdominal pain in the emergency department where they may be evaluated by physicians or surgeons with limited experience in bariatric surgery. While early consultations with surgeons with extensive experience in bariatric surgery are warranted, difficulties in reaching such colleagues in combination with pressure on a daily basis from various authorities, such as the legal system, the department of finance, and the public, to write prescriptions and order imaging examinations may in part explain the common use of CT scans in the evaluation of acute abdominal pain among these patients [21–25]. It is, however, important to emphasize that while CT scanning is generally the preferred radiological evaluation for SBO and IH, the sensitivity of this modality is approximately 83–85%, and it cannot replace the role of thorough clinical examinations and adequate patient histories [5, 8]. A tendency toward replacing thorough clinical evaluations with radiological evaluations may lead to a risk of missing certain cases and unnecessary radiation and thereby, an increase in the overall lifetime cancer risk.

The cancer risk associated with radiation exposure from any given CT scan can be estimated theoretically by either measuring or calculating the doses to the organ involved and then applying organ-specific cancer incidence/mortality data from atomic bomb survivors [9]. According to Gerber et al., there is an estimated risk of 0.05% for a fatal malignancy from a single abdominal CT scan at an effective dose of 10 mSv [26]. When comparing this risk to the risk of dying as a result of certain conditions or activities of everyday life, such as passive smoking (0.4–1%) or radon in homes (0.3%), or the natural incidence of fatal cancer (21.2% in the US population), the relative risk of carcinogenesis from one abdominal CT scan is low [26].

However, the concern regarding radiation-induced cancer due to CT scans is due to not only a single examination but rather the current rapid increase in CT usage and the overall effect of this increase. Individual cases of low risk applied to a fast-growing population may very well result in a potential public health issue in some years in the future. In our study, 132 patients underwent a total of 281 CT scans, which led to an overall radiation dose of 168 200 mGy cm for the entire group. This equals a collective dose of approximately 2800 manSv. Using the risk factor 5%/Sv for fatal cancer [9] would imply a prediction of 0.14 cases. While the individual risk is influenced by strong variations in radiosensitivity with age, sex, and BMI, as well as what part of the body is exposed and type of malignancy, this is not a number that can nor should be overlooked [9, 14]. It should be noted, however, that assessing risks based on collective dose is advised against by the International Commission on Radiological Protection (ICRP) due to large individual and statistical uncertainties [27]. Furthermore, when considering the risk of fatal cancer from radiation exposure after bariatric surgery, it is important to remember that the potential increase in risk remains low compared to the overall reduced cancer incidence after bariatric surgery [28].

The major strengths of this study include the randomized design with high rates of inclusion and follow-up after 5 years. Despite these strengths, the study has several limitations that must be acknowledged. First, although the original study was a registry-based nationwide multicenter RCT, the present post hoc analysis merely included a subpopulation from a single center. Local practices and resources along with geographical location may influence the use of imaging services. Therefore, the generalizability to other regions and countries may be limited. Second, as described previously, mesenteric defects closure is associated with a learning curve during which the risk for bowel obstruction in the early postoperative phase is increased [5, 29, 30]. This could have overestimated the use of CT scans for the group with closed mesenteric defects although only to a limited extent considering the low overall risk for this complication [5, 30]. Finally, imaging services other than CT, such as magnetic resonance imaging, ultrasound, or fluoroscopy, were not considered in this particular study. This may to some extent have narrowed the overall clinical picture of these patients and the range of complications they may have suffered.

## Conclusion

Almost half of the patients who underwent LRYGB underwent a CT scan for abdominal pain within 5 years of surgery. Closure of the mesenteric defects did not influence the use of CT for abdominal pain.
